# Identification of *Enterococcus faecalis* in a patient with urinary-tract infection based on metagenomic next-generation sequencing: a case report

**DOI:** 10.1186/s12879-020-05179-0

**Published:** 2020-07-02

**Authors:** Manshi Li, Fuhuo Yang, Yihan Lu, Weifeng Huang

**Affiliations:** 1 Department of Epidemiology, School of Public Health, Fudan University, Shanghai, 200032 China; 2 Dinfectome Inc., Shanghai, 200120 China; 3 Ministry of Education Key Laboratory of Public Health Safety (Fudan University), Shanghai, 200032 China; 4 Department of Intensive Care Medicine, The Sixth People’s Hospital, Shanghai Jiao Tong University, Shanghai, 200233 China

**Keywords:** Urinary tract infection (UTI), *Enterococcus faecalis*, Metagenomic next-generation sequencing (mNGS)

## Abstract

**Background:**

Urinary tract infection (UTI) caused by various pathogenic microorganisms is ubiquitous in the parts of the urinary system such as kidney, ureter, bladder, and urethra. Currently, clinical detection of UTI is mainly focused on urine culture; however, the diagnostic value of urine culture remains limited due to the time-consuming procedure and low detection rate, especially in patients who have used antibiotics. Generally, treatment for UTI relies on empirical medication rather than pathogen diagnosis, which leads to the inappropriate use of antimicrobial agents and a significant increase in resistant strains. Comparatively, metagenomic next-generation sequencing (mNGS) is capable of overcoming the disadvantages of clinical culture, and identifying pathogens for further treatment.

**Case presentation:**

A 33-year-old male patient was admitted to hospital with a high fever and chills. None of his autoimmune disease or thyroid function related indicators were positive, and he had no risk of endocarditis. His white blood cell count, C-reactive protein, procalcitonin, interleukin 6, and neutrophil proportion were markedly elevated. He was initially diagnosed as having an infection of unknown etiology. Since empirical treatment of Sulperazon and Metronidazole did not relieve his symptoms, both the blood and urine specimens were examined using traditional culture, serological testing, and mNGS assay. Traditional culture and serological testing produced negative results, while the mNGS assay revealed the presence of a potential pathogen, *Enterococcus faecalis*, in the urine specimen, which was further confirmed by both Sanger sequencing and qPCR analysis. A CT scan of the patient’s whole abdomen showed stones in the right kidney. Once targeted antibiotic therapy was administered, the patient recovered quickly.

**Conclusions:**

Our case illustrated that mNGS, as a novel culture-independent approach, demonstrated the capability of rapid, sensitive, and accurate pathogen identification. Furthermore, this technology provides strong support for guiding clinicians to determine appropriate treatment.

## Background

Urinary tract infection (UTI) is one of the most prevalent community-acquired and hospital-acquired infections. It has been indicated that UTI is caused by various pathogenic microorganisms, among which the majority are *Escherichia coli*, *Klebsiella pneumoniae*, *Proteus mirabilis*, *Enterococcus faecalis,* and *Staphylococcus saprophyticus* [1]. UTI mainly occurs in women of childbearing age [2], elders [3], and people with low immunity and urinary tract abnormalities. Antibiotic treatment is usually the priority treatment strategy for UTI patients [4, 5] However, due to the recent serious antibiotic abuse, pathogen resistance to antibiotics has been increased dramatically, which further increases disease burden [1]. Therefore, it is crucial to rapidly and accurately identify the pathogens of UTI, and then optimize medical therapy.

The gold standard for diagnosis of UTI remains quantitative urine culture, though various pathogen diagnostic methods are available [6, 7]. However, standard laboratory quantitative urine culture for bacteria usually takes at least 18 h, which indicates possible difficulty in the diagnosis 24 to 48 h after onset [6, 8]. In addition, due to previous use of antibiotics before admission to hospitals, the sensitivity of traditional culture in detecting pathogens remains limited, especially in patients with sepsis [8]. Therefore, incapability of obtaining a targeted and timely diagnosis might lead to inappropriate treatment, antibiotic resistance, and increased medical costs. Moreover, as many viruses and parasites are difficult or impossible to culture traditionally, diagnosis can be performed by serological and molecular methods based on the detection of specific antigens, antibodies, or genes. However, prior judgment by clinicians remains important and necessary for the diagnosis [9, 10].

Metagenomic next-generation sequencing (mNGS) is an emerging detection technique for supporting pathogen diagnosis without the requirement of preclinical prediction or culture. mNGS is capable of identifying novel or rare pathogens by determining all sequences of microbial genomes in clinical specimens within 24–48 h [11], which presents significant advantages in conditional pathogen infection and mixed infection. This further facilitates accurate diagnosis and optimal therapy. Herein, we have presented a case of UTI with *Enterococcus faecalis* (*E. faecalis*), in which traditional culture was negative but mNGS provided a positive finding. Consequently, the patient received targeted linezolid therapy and then recovered.

## Case presentation

**Fig. 1:**
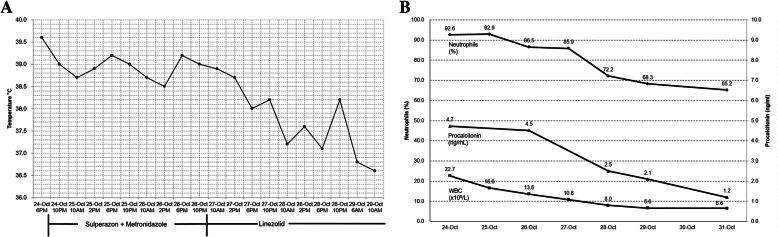
Dynamic monitoring of the patient’s body temperature and inflammatory markers. **a** Trend of body temperature by treatment. **b** Trend of inflammatory markers by routine blood testing, including WBC(× 10^9^/L), neutrophil proportion(%) and procalcitonin (ng/mL) over time

A 33-year-old male was admitted to hospital due to having a high fever with a lower abdominal pain for 1 day. Before arriving at the hospital, the patient developed chills, and his body temperature rose to 39.6 °C. He was administered cold medicine to be taken orally and advised to rest for several hours; however, his symptoms were not significantly relieved, neither did his body temperature drop (Fig. 1a).

Laboratory testing was performed. Routine blood testing showed that the white blood cell (WBC) count was 22.69 × 10^9^/L with a proportion of 92.6% being neutrophils. The procalcitonin (PCT) level was elevated to 4.52 ng/mL, C-reactive protein (CRP) reached 170 mg/L, and serum interleukin 6 was 34.74 pg/mL (Fig. 1b). Liver and kidney function was basically normal. Routine autoimmune diseases-related indicators were negative, including anti-cardiac antibodies, anti-SMA, anti-AMA, anti-MPO antibodies, anti-RNP/Sm antibodies, anti-SS-A antibodies, anti-SS-B antibodies, anti-Scl-70 antibodies, anti-PM-Scl antibodies, anti-Jo-1 antibodies, and anti-dsDNA antibodies. In addition, NK and T cell subpopulations, complements C3 and C4, anti-cyclic citrullinated peptide antibody, glucose 6-phosphate isomerase, and thyroid function-related indicators including triiodothyronine, thyroxine, free triiodothyronine, free thyroxine, thyroid stimulating hormone, and parathyroid hormone, were normal. Indicators within routine urine testing were normal, including WBC. An echocardiography also ruled out endocarditis. Based on previous clinical experience, the initial diagnosis was determined as infection of unknown etiology.

**Fig. 2:**
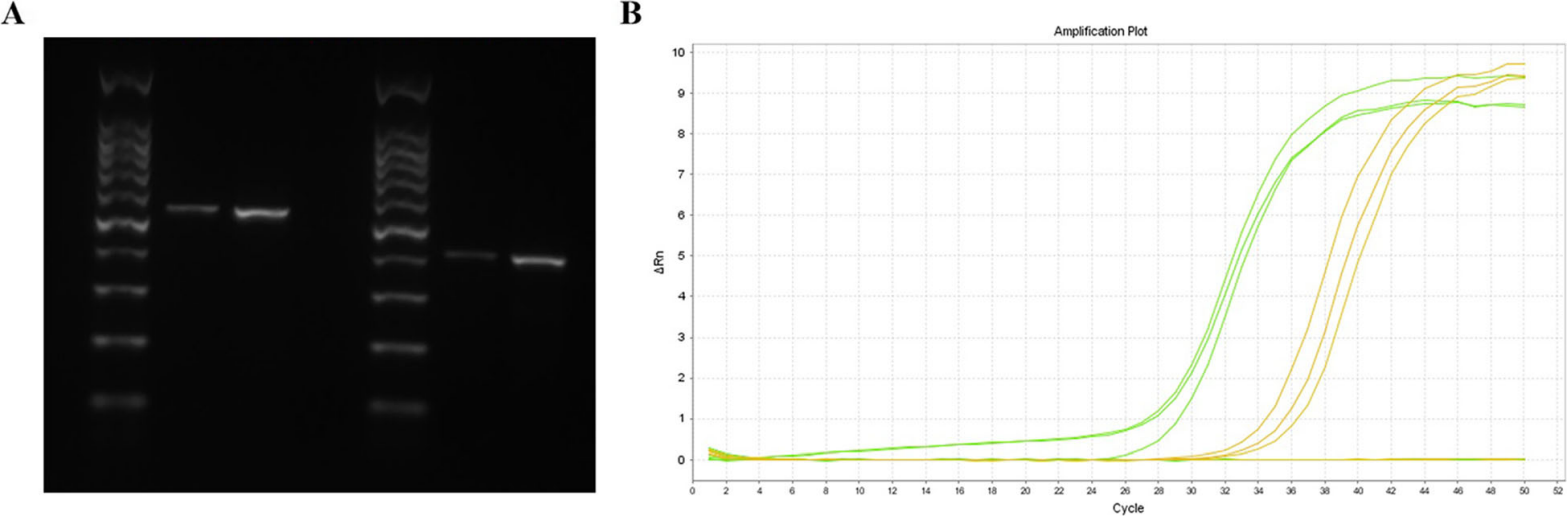
PCR electrophoretogram and qPCR analysis of the *Enterococcus faecalis.***a** Electrophoretogram identified *Enterococcus faecalis*. **b** Amplification curve of the real-time qPCR confirmed the *Enterococcus faecalis*

An initial treatment of Sulperazone (3 g/8 h iv) and Metronidazole (0.5 g/12 h iv) had been empirically administrated for 3 days. The patient’s chills disappeared, but he still had an intermittent fever with a peak of 38.8 °C. Serological testing was performed, including antibodies to respiratory syncytial virus, adenovirus, influenza A and B viruses, *Chlamydia pneumoniae*, *Mycoplasma pneumoniae,* and *Legionella pneumophila*; however, all of them were negative. Following this, both his peripheral blood and urine specimens were examined to investigate possible etiology using traditional culture and mNGS (Dinfectome Inc., Shanghai, China). Both aerobic and anaerobic bacterial cultures in the peripheral blood and urine specimens were negative after 5 days’ culture. However, within 24 h, mNGS revealed a total of 27,831,980 single-end reads in the genomic DNA of the urine specimen, of which 163 reads were *E. faecalis* (Table 1). Subsequently, sanger sequencing and PCR electrophoretogram confirmed identification of *E. faecalis* in the urine specimen (Fig. 2a, Table S1 and Figure S1). QPCR assay of *E. faecalis* showed a Ct value of 32, and a positive control Ct value of 26 (Fig. 2b).

**Table 1:** Pathogenic microorganisms detected by using next-generation sequencing in original and follow-up urine specimen

Original urine specimen			Follow-up urine specimen		
Species	Category	Number of detected reads	Species	Category	Number of detected reads
*Homo sapiens*	Human	26,263,435	*Homo sapiens*	Human	15,341,902
*Enterococcus faecalis*	Bacteria	163	*Enterococcus faecalis*	Bacteria	2
*JC polyomavirus*	Virus	91	*JC polyomavirus*	Virus	202
*Human herpesvirus 6*	Virus	3	*Human herpesvirus 6*	Virus	1
*Malassezia globosa*	fungus	77	*Streptococcus pneumoniae*	Bacteria	129
*Actinomyces neuii*	Bacteria	32	*Streptococcus mitis*	Bacteria	59
*Staphylococcus haemolyticus*	Bacteria	8	*Ureaplasma parvum*	Bacteria	56
*Cutibacterium avidum*	Bacteria	6	*Streptococcus pseudopneumoniae*	Bacteria	51
Other species: *Meiothermus ruber*, *Comamonas testosteroni, Yarrowia lipolytica, Acinetobacter ursingii,* etc.			*Streptococcus oralis*	Bacteria	26
			*Staphylococcus lugdunensis*	Bacteria	3
			Other species: *Xanthomonas campestris, Cutibacterium acnes, Delftia acidovorans, Acinetobacter ursingii, Meiothermus ruber*, etc.

**Fig. 3:**
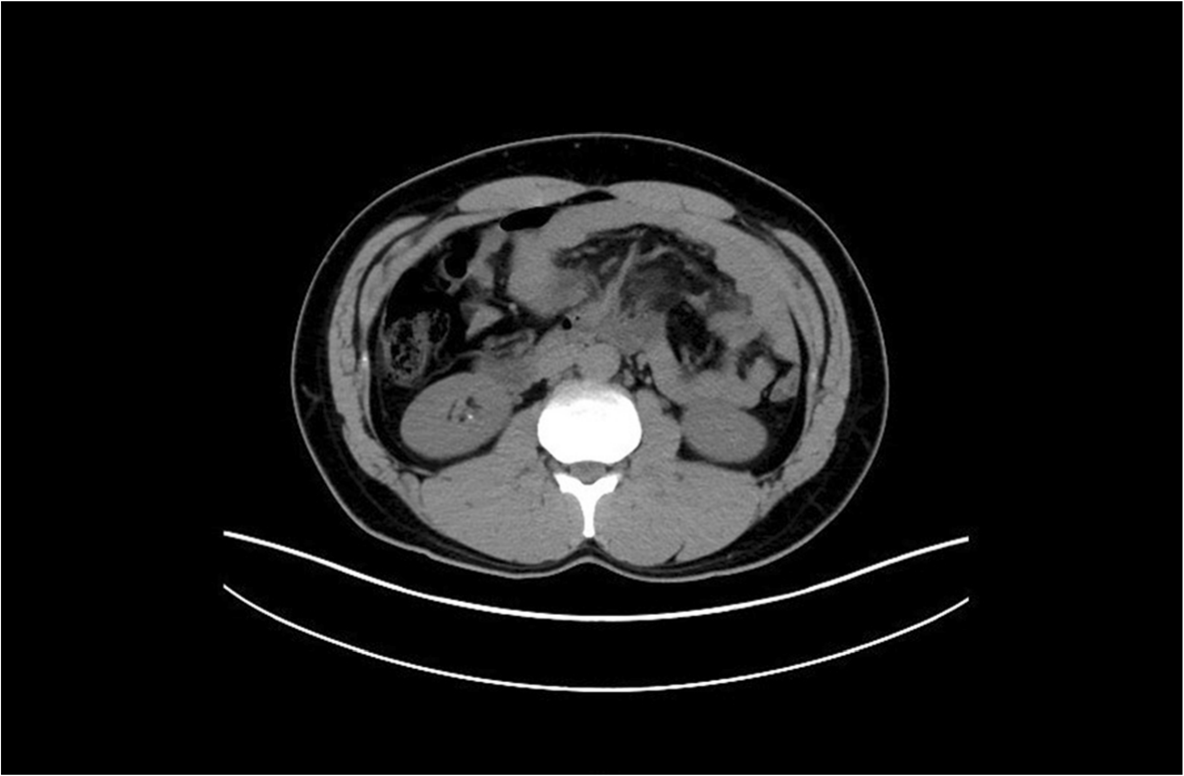
Whole abdominal CT of patients. CT image showed small stones in his right kidney

Furthermore, from the patient’s medical history, we learned that he had a mesenteric cyst in 2015. His whole abdominal CT showed a cystic mass in the mesenteric space of the abdominal cavity with a maximum cross-section of 18 × 7 cm and small stones in his right kidney (Fig. 3). This suggested that the patient had a UTI, and the *E. faecalis* may be the cause. We then replaced Sulperazon with Linezolid (0.6 g/12 h po) for his therapy. Consequently, the patient’s temperature dropped and inflammation indicators (WBC, PCT, and CRP) gradually returned to normal (Fig. 1). Three days later, the patient was discharged without any complications. In the 11th day of the patient’s follow-up period, a urine sample was taken again for mNGS assay. A total of 15,974,870 reads were obtained, and the number of *E. faecalis* decreased to 2 unique reads (Table 1). The relative abundance, the proportion of a detected microbe of total reads, was calculated for comparison. The relative abundance of *E. faecalis* was decreased from 0.11148856 to 0.00274632, suggesting effective treatment.

The experiment procedure for mNGS detection is as follows: DNA from urine samples and blood samples is extracted using Tiangen Magnetic DNA Kit (Tiangen). The extracted DNA was fragmented ultrasonically to yield 150–300 bp fragments. Libraries were prepared using the KAPA library preparation kit (KAPA Biosystem). After quantitation and qualification, the libraries were 75 bp single-end sequenced on Illumina NextSeq 550Dx (Illumina). An in-house developed bioinformatics pipeline was used for pathogen identification. High-quality sequencing data were generated by removing adapter, low quality bases, duplicated reads, and short (length < 36 bp) reads. Human host sequences were identified by mapping to human reference genome (hs37d5) using bowtie2 software. Reads that could not be mapped to the human genome were retained and aligned with microorganism genome database for pathogen identification. Our microorganism genome database contained genomes or scaffolds of bacteria, fungi, viruses and parasites related to human infectivity (download from ftp://ftp.ncbi.nlm.nih.gov/genomes/genbank/). Reads were verified using blastn in the NT database and species with reads > = 3 were reported. The obtained sequencing data were submitted to the Sequence Read Archive of the National Center for Biotechnology Information (NCBI) with the accession number SRR11624506, SRR11624507 and SRR11624508.

## Discussion and conclusions

Here, we reported a case in which mNGS facilitated clinicians rapidly and accurately identifying *E. faecalis* in a patient with UTI. This patient was admitted to hospital due to high fever and chills. Traditional culture and serological testing did not determine possible infection etiology, while mNGS identified *E. faecalis* in the urine specimen. Combined with his clinical characteristics, his diagnosis was confirmed to be a UTI.

*Enterococci* are a class of bacteria typically found in the human gastrointestinal tract, mouth or vagina. *E. faecalis* and *E. faecium* are the two most common enterococci isolated in clinical samples [12]. A survey indicated that *E. faecalis* can be identified in about 80% of human infections [13]. It is known to be one of the main causes of human UTI worldwide [1]. In recent years, the incidence of UTI caused by *E. faecalis* has been estimated to be five times than that of *E. faecium* [14]. In our case, the patient had coexisting kidney stones that are common in the urinary system. Theoretically, once the bacteria has invaded the urinary tract and contributed to urinary stone formation, it triggers UTI easily, and further develops chronic pyelonephritis [15]. In addition to *E. faecalis*, JC polyomavirus and human herpesvirus 6 were detected in the patient’s urine, but were not considered to have caused the patient’s UTI as both of them usually cause asymptomatic persistent or latent infection [16, 17]. Therefore, clinicians need to have a good professional knowledge when interpreting mNGS findings. We employed mNGS to evaluate the treatment effects by comparing unique reads before and after treatment. Once the treatment was switched to Linezolid therapy, the patient recovered immediately after 3 days. In addition, the unique reads of *E. faecalis* in our patient declined dramatically to 2 within 2 weeks, suggesting *E. faecalis* may be the cause of the UTI, and further confirmed the treatment effects.

mNGS, as a non-biased method for rapid diagnosis of pathogens, overcomes many of the deficiencies of traditional detection methods, and directly performs DNA or RNA sequencing on samples [11, 18, 19], which is increasingly being applied in clinical laboratories. Compared with other diagnostic methods, mNGS has many advantages, but also some limitations. A prominent advantage of mNGS is that it is a completely unbiased technology that can replace many target pathogen tests with a single mNGS assay, which targets all pathogens (bacteria, fungi, viruses, and parasites) in the specimens without the need for doctors to prejudge. Therefore, for the diagnosis and identification of some rare or unknown pathogens, mNGS has comparative advantages. In addition, mNGS is appropriate for a variety of specimen types, including peripheral blood, cerebrospinal fluid, tissue, sputum, and bronchoalveolar lavage, and could be implemented in the clinical practices of sepsis, immunosuppressive host with severe infection, severe pulmonary infection, rare or new pathogen infection, and other infectious diseases [20, 21]. It would broaden the application of mNGS in the clinical practice and further bedside decision making. Furthermore, mNGS can greatly reduce the turnaround time for pathogen identification, and is more sensitive than the cultivation method [19, 22]. Routine culture is a gold standard method for organism identification, but their sensitivity is often low due to prior antibiotics and antifungals exposure [23]. Bacterial and yeast cultivation is generally time consuming, and fastidious organisms are not easy to culture. Additionally, for the identification of viruses or parasites, the role of cultivation is often limited [24]. Therefore, multiple infections are often easily overlooked, and the detection of pathogens in unexplained fever patients is even more difficult for clinical diagnosis. mNGS has the capability to avoid the limitations of traditional culture tests allowing for quickly and effectively identifying the known and unknown pathogens in the specimen within 24–48 h [11] and improving the clinical diagnosis rate [20]. Many successful cases and studies have proved the great potential of mNGS in infectious disease diagnostics. Most articles are published as case reports, such as identifying *Leptospirosis* [25], *Bornavirus* [26], *Chlamydia psittaci* [27], varicella-zoster virus [28], *Parvimonas micra* [29], and 2019-nCoV [30] in the specimens using mNGS, compared to negative findings using traditional methods. In some multi-center or multi-sample research, mNGS has demonstrated better diagnostic performance for pathogens compared to culture or other methods for different disease types. For the diagnosis of sepsis [31], severe pneumonia [32], encephalitis and meningitis [33], suspected focal infection [22], and infection caused by immunodeficiency after transplantation [18], mNGS can significantly improve the clinical diagnosis rate, and the sensitivity of pathogen identification is significantly higher than traditional microbial diagnostic methods. However, there are still many practical problems in the clinical application of mNGS. It is not easy to distinguish between colonizing bacteria, background bacteria and pathogenic bacteria among the various species detected [21]. In our case, the detected microorganisms might be from the environment (*Meiothermus* and *Comamonas*), reagents (*Yarrowia* and *Acinetobacter*), consumables, or the surface of the patient’s skin (*Staphylococcus lugdunensis* and *Malassezia*), or elsewhere. Therefore, it is necessary to set up negative controls on the same batch of samples during the experiment and excluding background pathogens through the establishment of a large sample database in the early stage. Another disadvantage of mNGS is the amplification of host nucleic acids. More than 99% of reads generated by sample sequencing are from human hosts [19], and microorganisms account for only a small proportion. Therefore, sequencing all nucleic acids reduces the sensitivity of pathogen identification. The host nucleic acids can be depleted by certain methods during wet experiments [34–36]. Reducing the proportion of human-derived nucleic acid sequences can increase the data volume of microorganisms to a certain extent and increase sensitivity.

In conclusion, our case illustrated the potential application of mNGS in detecting pathogenic microorganisms in samples which were not detected by traditional culture and serological testing. This study suggests that mNGS could be implemented for monitoring the progress of the disease and evaluating therapy effects. It is believed that in the near future, as the cost of sequencing continues to decline, mNGS will be more and more widely used in clinics, benefiting more doctors and patients.

## Supplementary information

**Additional file 1: Table S1.** Primers used in polymerase chain reaction. **Figure S1.** Electrophoretogram of PCR identified *Enterococcus faecalis*. On the right side of the figure, two sets of primers are used for PCR amplification. Urine represents the patient’s first urine sample. PTC represents positive template control. NTC represents negative template control. L100 represents DNA ladder.

## Data Availability

The data supporting the conclusions discussed in this article are included within the article. The datasets used and/or analyzed in the current study are available in NCBI with the accession number SRR11624506, SRR11624507 and SRR11624508. SRR11624507 and SRR11624508 are the sequencing data of urine and blood samples detected for the first time, and SRR11624506 is the urine sample sequencing data during the second follow-up.

## Abbreviations

UTI: Urinary tract infection
mNGS: Metagenomic next-generation sequencing
*E. faecalis*: *Enterococcus faecalis*
WBC: White blood cell
PCT: Procalcitonin
CRP: C-reactive protein
SSSIs: Skin structure infections

## References

[1] Flores-Mireles AL, Walker JN, Caparon M, Hultgren SJ (2015). Urinary tract infections: epidemiology, mechanisms of infection and treatment options. Nat Rev Microbiol.

[2] Matuszkiewicz-Rowinska J, Malyszko J, Wieliczko M (2015). Urinary tract infections in pregnancy: old and new unresolved diagnostic and therapeutic problems. Arch Med Sci.

[3] Schaeffer AJ, Nicolle LE (2016). Urinary tract infections in older men. N Engl J Med.

[4] Tandogdu Z, Wagenlehner FM (2016). Global epidemiology of urinary tract infections. Curr Opin Infect Dis.

[5] Foxman B (2010). The epidemiology of urinary tract infection. Nat Rev Urol.

[6] Najeeb S, Munir T, Rehman S, Hafiz A, Gilani M, Latif M (2015). Comparison of urine dipstick test with conventional urine culture in diagnosis of urinary tract infection. J Coll Physicians Surg Pak.

[7] Schmiemann G, Kniehl E, Gebhardt K, Matejczyk MM, Hummers-Pradier E (2010). The diagnosis of urinary tract infection: a systematic review. Dtsch Arztebl Int.

[8] Maxson T, Mitchell DA (2016). Targeted treatment for bacterial infections: prospects for pathogen-specific antibiotics coupled with rapid diagnostics. Tetrahedron.

[9] Kaiser L (2013). Counterpoint: is the era of viral culture over in the clinical microbiology laboratory?. J Clin Microbiol.

[10] Balsalobre-Arenas L, Alarcon-Cavero T (2017). Rapid diagnosis of gastrointestinal tract infections due to parasites, viruses, and bacteria. Enferm Infecc Microbiol Clin.

[11] Miao Q, Ma Y, Wang Q, Pan J, Zhang Y, Jin W, Yao Y, Su Y, Huang Y, Wang M (2018). Microbiological Diagnostic Performance of Metagenomic Next-generation Sequencing When Applied to Clinical Practice. Clin Infect Dis.

[12] Goel V, Kumar D, Kumar R, Mathur P, Singh S (2016). Community acquired Enterococcal urinary tract infections and antibiotic resistance profile in North India. J Lab Physicians.

[13] Huycke MM, Sahm DF, Gilmore MS (1998). Multiple-drug resistant enterococci: the nature of the problem and an agenda for the future. Emerg Infect Dis.

[14] Kline KA, Lewis AL (2016). Gram-Positive Uropathogens, Polymicrobial Urinary Tract Infection, and the Emerging Microbiota of the Urinary Tract. Microbiol Spectr.

[15] Borghi L, Nouvenne A, Meschi T (2012). Nephrolithiasis and urinary tract infections: 'the chicken or the egg' dilemma?. Nephrol Dial Transplant.

[16] Divers J, Langefeld CD, Lyles DS, Ma L, Freedman BI (2019). Protective association between JC polyoma viruria and kidney disease. Curr Opin Nephrol Hypertens.

[17] Caserta MT, Mock DJ, Dewhurst S (2001). Human herpesvirus 6. Clin Infect Dis.

[18] Lewandowska DW, Schreiber PW, Schuurmans MM, Ruehe B, Zagordi O, Bayard C, Greiner M, Geissberger FD, Capaul R, Zbinden A (2017). Metagenomic sequencing complements routine diagnostics in identifying viral pathogens in lung transplant recipients with unknown etiology of respiratory infection. PLoS One.

[19] Simner PJ, Miller S, Carroll KC (2018). Understanding the promises and hurdles of metagenomic next-generation sequencing as a diagnostic tool for infectious diseases. Clin Infect Dis.

[20] Chiu CY, Miller SA (2019). Clinical metagenomics. Nat Rev Genet.

[21] Gu W, Miller S, Chiu CY (2019). Clinical metagenomic next-generation sequencing for pathogen detection. Annu Rev Pathol.

[22] Zhang HC, Ai JW, Cui P, Zhu YM, Hong-Long W, Li YJ, Zhang WH (2019). Incremental value of metagenomic next generation sequencing for the diagnosis of suspected focal infection in adults. J Inf Secur.

[23] Limmathurotsakul D, Jamsen K, Arayawichanont A, Simpson JA, White LJ, Lee SJ, Wuthiekanun V, Chantratita N, Cheng A, Day NP (2010). Defining the true sensitivity of culture for the diagnosis of melioidosis using Bayesian latent class models. PLoS One.

[24] Laupland KB, Valiquette L (2013). The changing culture of the microbiology laboratory. Can J Infect Dis Med Microbiol.

[25] Wilson MR, Naccache SN, Samayoa E, Biagtan M, Bashir H, Yu G, Salamat SM, Somasekar S, Federman S, Miller S (2014). Actionable diagnosis of neuroleptospirosis by next-generation sequencing. N Engl J Med.

[26] Hoffmann B, Tappe D, Hoper D, Herden C, Boldt A, Mawrin C, Niederstrasser O, Muller T, Jenckel M, van der Grinten E (2015). A variegated squirrel Bornavirus associated with fatal human encephalitis. N Engl J Med.

[27] Gu L, Liu W, Ru M, Lin J, Yu G, Ye J, Zhu ZA, Liu Y, Chen J, Lai G (2020). The application of metagenomic next-generation sequencing in diagnosing chlamydia psittaci pneumonia: a report of five cases. BMC Pulm Med.

[28] Fang M, Weng X, Chen L, Chen Y, Chi Y, Chen W, Hu Z (2020). Fulminant central nervous system varicella-zoster virus infection unexpectedly diagnosed by metagenomic next-generation sequencing in an HIV-infected patient: a case report. BMC Infect Dis.

[29] Huang Z, Zhang C, Li W, Fang X, Wang Q, Xing L, Li Y, Nie X, Yang B, Zhang W (2019). Metagenomic next-generation sequencing contribution in identifying prosthetic joint infection due to Parvimonas micra: a case report. J Bone Jt Infect.

[30] Chen L, Liu W, Zhang Q, Xu K, Ye G, Wu W, Sun Z, Liu F, Wu K, Zhong B (2020). RNA based mNGS approach identifies a novel human coronavirus from two individual pneumonia cases in 2019 Wuhan outbreak. Emerg Microbes Infect.

[31] Long Y, Zhang Y, Gong Y, Sun R, Su L, Lin X, Shen A, Zhou J, Caiji Z, Wang X (2016). Diagnosis of Sepsis with cell-free DNA by next-generation sequencing technology in ICU patients. Arch Med Res.

[32] Xie Y, Du J, Jin W, Teng X, Cheng R, Huang P, Xie H, Zhou Z, Tian R, Wang R (2019). Next generation sequencing for diagnosis of severe pneumonia: China, 2010-2018. J Inf Secur.

[33] Wilson MR, Sample HA, Zorn KC, Arevalo S, Yu G, Neuhaus J, Federman S, Stryke D, Briggs B, Langelier C (2019). Clinical metagenomic sequencing for diagnosis of meningitis and encephalitis. N Engl J Med.

[34] Hasan MR, Rawat A, Tang P, Jithesh PV, Thomas E, Tan R, Tilley P (2016). Depletion of human DNA in spiked clinical specimens for improvement of sensitivity of pathogen detection by next-generation sequencing. J Clin Microbiol.

[35] Auburn S, Campino S, Clark TG, Djimde AA, Zongo I, Pinches R, Manske M, Mangano V, Alcock D, Anastasi E (2011). An effective method to purify plasmodium falciparum DNA directly from clinical blood samples for whole genome high-throughput sequencing. PLoS One.

[36] Feehery GR, Yigit E, Oyola SO, Langhorst BW, Schmidt VT, Stewart FJ, Dimalanta ET, Amaral-Zettler LA, Davis T, Quail MA (2013). A method for selectively enriching microbial DNA from contaminating vertebrate host DNA. PLoS One.

