# Supplementary material for: Identification of Enterococcus faecalis in a patient with urinary-tract infection based on metagenomic next-generation sequencing: a case report
Source: BMC Infect Dis. 2020 Jul 2;20:467. doi: 10.1186/s12879-020-05179-0 (PMC7330266; doi:10.1186/s12879-020-05179-0)
Supplement: Supplementary file 1 — Additional file 1: Table S1. Primers used in polymerase chain reaction. Figure S1. Electrophoretogram of PCR identified Enterococcus faecalis. On the right side of the figure, two sets of primers are used for PCR amplification. Urine represents the patient’s first urine sample. PTC represents positive template control. NTC represents negative template control. L100 represents DNA ladder. [file 12879_2020_5179_MOESM1_ESM.doc]

**Supplementary information**

**Table S1. Primers used in polymerase chain reaction**

| Primers | Primers sequence (5’-3’) | Amplicon (bp) |
| --- | --- | --- |
| E.fae-5F | GCCACCGCCATACGGATAA | 525 |
| E.fae-5R | AGTCCCACCGCAAGCATA | 525 |
| E.fae-6F | CTGGGAGTAACGCGGATAT | 402 |
| E.fae-6R | CTGTCGCAACGGCAAGTA | 402 |


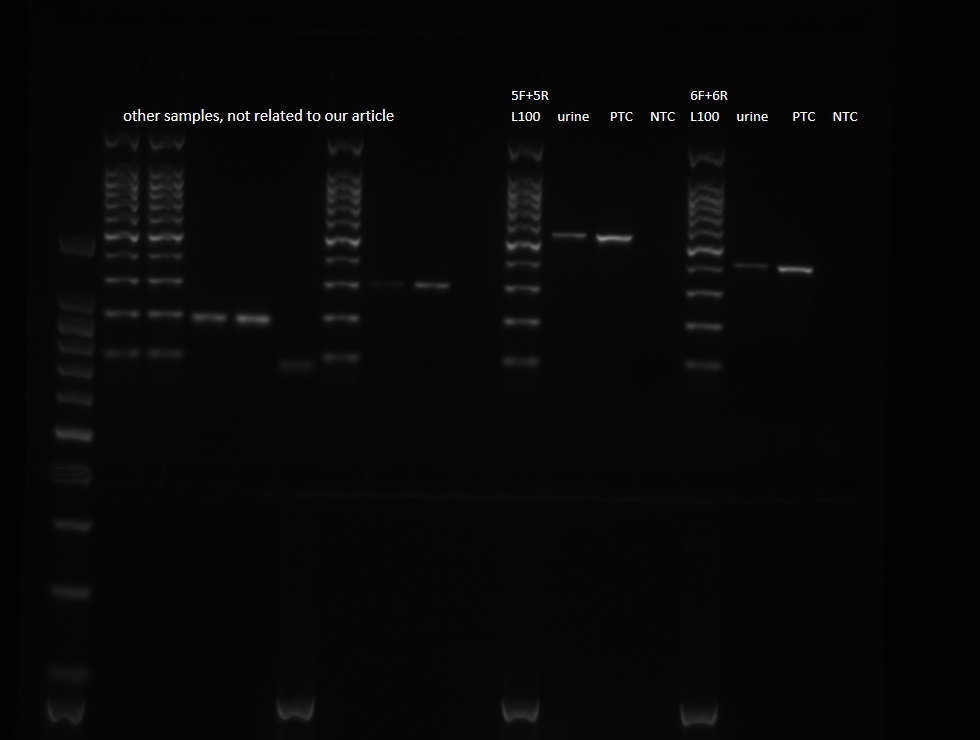


Figure S1. Electrophoretogram of PCR identified *Enterococcus faecalis*. On the right side of the figure, two sets of primers are used for PCR amplification. Urine represents the patient's first urine sample. PTC represents positive template control. NTC represents negative template control. L100 represents DNA ladder.
